# Is the rate of postoperative complications following laparoscopy-assisted gastrectomy higher in elderly patients than in younger patients?

**DOI:** 10.1186/1477-7819-12-97

**Published:** 2014-04-16

**Authors:** Ki-Han Kim, Min-Chan Kim, Ghap-Joong Jung

**Affiliations:** 1 Department of Surgery, Dong-A University College of Medicine, 3-1 Dongdaeshin-Dong, Seo-Gu, Busan 602-715, Korea

**Keywords:** Age, Gastrectomy, Laparoscopy, Postoperative complication

## Abstract

**Background:**

With an increase in life expectancy, very elderly patients are presenting with gastric cancer more commonly than ever. The present study retrospectively analyzed the surgical outcomes of laparoscopy-assisted gastrectomy for gastric cancer in the young, elderly, and very elderly age groups.

**Methods:**

The study group consisted of 1,055 patients who underwent laparoscopy-assisted gastrectomy between February 2002 and December 2012. We divided these patients into three groups; group 1 (young age, <65 years), group 2 (elderly age, 65–74 years), and group 3 (very elderly age, ≥75 years).

**Results:**

There were statistical differences in the rates of postoperative complications among the three groups (*P* = 0.008). However, when assessed according to the severity of postoperative complications based on the Clavien-Dindo classification, there was no statistical difference among the three groups (*P* = 0.562).

**Conclusions:**

Laparoscopy-assisted gastrectomy for gastric cancer can be performed in very elderly patients. In analyzing studies of elderly patients with postoperative complications following the procedure, not only should the rate of postoperative complications be taken into consideration, but also the severity of any postoperative complications.

## Background

According to the annual report of the Korea National Statistical Office in 2007, citing figures from the World Health Organization, the proportion of elderly people aged 65 years or more was 9.9% [1]. By 2018, the proportion of people older than 65 years in Korea is expected to exceed 14% [2]. Life expectancy is currently 74.4 years for men and 81.8 years for women [3]. Korea has one of the highest incidences of gastric cancer in the world, with the elderly, who are most likely to be affected by gastric cancer, representing the fastest growing group of the Korean population. The incidence of early gastric cancer, which is considered an appropriate indication for laparoscopy-assisted gastrectomy (LAG), is increasing in Korea because of recent improvements in early diagnosis and a well-developed mass-screening program [4,5]. As the mean age of the general population increases, an increase in numbers of elderly patients with gastric cancer can be expected, all of whom will be candidates for gastric resection.

LAG has been reported to be a less invasive surgical technique than open gastrectomy, with advantages that include good cosmetic effect, improved quality of life, minimal degree of pain, shorter hospital stay, early rehabilitation, and early return to social activity [6-8]. However, elderly patients are at high risk of intra- and post-operative complications owing to the high prevalence of comorbidities and decreased functional reserve in these patients. Many retrospective studies have evaluated gastrectomy in elderly patients with regard to feasibility and safety [9-11]. Several studies have also evaluated patients over 85 years old [12,13].

In this study, the authors aimed to investigate whether the use of LAG for gastric cancer can be extended to very elderly patients. In addition, the clinicopathological characteristics and surgical outcomes of gastric cancer were compared among three groups of patients (young, elderly, and very elderly) to determine the feasibility of LAG for very elderly patients.

## Methods

### Patient collection

From February 2002 through to December 2012, 1,055 consecutive patients who underwent LAG with lymph node dissection were enrolled in this study. In the initial early period of the study, the indication for LAG for gastric cancer was cT1N0M0. In 2006, the indication for LAG was extended to T2N1M0. Our surgical technique employed for laparoscopy-assisted distal gastrectomy has been described in detail in a previous report [14].

We gave information to the patients and obtained written consent from them for their operations. All patients underwent pre-operative evaluation, including gastrofiberoscopy and computed tomography of the abdomen and pelvis. Those over 70 years of age, or who had heart problems, were evaluated with echocardiography. At-risk cases with pulmonary conditions underwent pulmonary function testing. In some cases, endoscopic ultrasonography was performed to assess the depth of cancer invasion.

According to the 2007 annual report of the Korea National Statistical Office, elderly patients are defined as those aged 65 years more [1]. Therefore, for the purposes of comparison, we divided enrolled patients into three age groups: group 1 (young age, <65 years), group 2 (elderly age, 65–74 years), and group 3 (very elderly age, ≥75 years). Clinicopathological characteristics and surgical outcomes of gastric cancer were compared among the three groups.

### Data collection

We prospectively collected data from our gastric cancer database and retrospectively reviewed the medical records. Clinicopathologic features such as age, gender, body mass index (BMI), comorbidity disease, tumor size, histologic type, tumor location, gastric resection type, reconstruction type, T stage, N stage, retrieved lymph node, and TNM stage were compared among the three groups. In addition, post-operative outcomes, hospital course, post-operative morbidity, and post-operative mortality were evaluated.

In this study, gastric cancer stage was classified according to the 7th edition of the American Joint Committee on Cancer staging criteria [15]. The patients enrolled in this study underwent standard D2 or above, according to the 2010 Japanese gastric cancer treatment guidelines (ver.3) [16]. All the values were expressed as means ± standard deviations.

All follow-up patients were monitored post-operatively by a routine check for blood tests, tumor markers (alpha-fetoprotein, carcinoembryonic antigen, and carbohydrate antigen 19-9), chest radiography, endoscopy, and computed tomography every 6 months for 2 years, then every year for the next 3 years.

### Definition of complications

Operative mortality was defined as death within 30 days of surgery. Morbidities were defined as complications that required additional treatment after surgery. Initially, we collected data on postoperative complications among the three groups. Next, for standardization, these complications were graded according to the Clavien-Dindo classification, which was adopted using objective criteria to assess the severity and incidence of postoperative complications [17,18].

### Standardized postoperative protocol

All patients were managed routinely by a standardized peri-operative protocol, as follows: i) no nasogastric intubation or pre-operative mechanical bowel preparation; ii) minimal spillage of gastric contents; iii) use of two closed suction drains; iv) in case of subtotal gastrectomy, sips of water 48 h post-operatively; v) in case of total gastrectomy, sips of water 72 h post-operatively; vi) a clear liquid diet for 3 days (subtotal gastrectomy) or 4 days (total gastrectomy) post-operatively; vii) hospital discharge 7 or 8 days after a soft diet with no abnormal clinical symptoms.

### Statistical analysis

The data were analyzed by Student’s *t*-test or Mann–Whitney U-test, in addition to the χ^2^ test, and Fisher’s exact test was used using SPSS version 18.0 (SPSS, Chicago, Ill, USA). Statistical significance was assumed for *P* values <0.05.

## Results

### Patient demographics

The clinicopathologic features among the three groups are listed in Table 1. There were no statistical differences in gender, BMI, tumor size, tumor location, N stage, and TNM stage. However, there was statistical significance with comorbidity, age, histologic type, and T stage (*P* <0.001, *P* <0.001, and *P* <0.001), respectively. In group 3, there were more cases with advanced gastric cancer (*P* = 0.032).

**Table 1:** Clinicopathological features among the three groups

	**Group 1 (n = 672)**	**Group 2 (n = 285)**	**Group 3 (n = 98)**	***P *value**
Age (year)^*^	51.3 ± 8.9	69.3 ± 2.9	77.6 ± 2.6	<0.001
**Gender (%)**				0.203
Male	398 (59.2)	186 (65.3)	58 (59.2)	
Female	274 (40.8)	99 (34.7)	40 (40.8)	
**BMI (kg/m**^**2**^)^*^	23.5 ± 2.8	23.2 ± 3.0	23.0 ± 3.2	0.253
**Comorbidity (number,%)**	171 (25.4)	141 (49.5)	66 (67.3)	<0.001
Hypertension^**^	105	43	24	
Diabetes^**^	46	25	6	
Heart disease^**^	20	10	3	
Pulmonary disease^**^	10	12	2	
Liver disease^**^	28	6	3	
Kidney disease^**^	2	1	0	
Others	11	8	4	
Size of main lesion (mm)^*^	2.8 ± 1.9	2.6 ± 1.6	3.0 ± 2.2	0.088
**Histologic type**				<0.001
Well differentiated	129	98	26	
Moderately differentiated	192	103	37	
Poorly differentiated	253	71	26	
Signet ring cell	82	5	2	
Other	16	8	7	
**Tumor location**				0.283
Upper	39	17	5	
Middle	211	79	21	
Lower	422	189	72	
T stage^***^(%)				0.032
EGC	538 (80.1)	232 (81.4)	68 (69.4)	
AGC	134 (19.9)	53 (18.6)	30 (30.6)	
N stage^***^				0.260
N0	573	249	79	
N1–N3	99	36	19	
Stage^***^				0.084
IA	534	228	67	
IB	41	22	11	
IIA	32	18	9	
IIB	25	11	5	
IIIA	14	4	4	
IIIB	20	2	2	
IIIC	6	0	0	

*All values are the means and standard deviations; **Combined number; ***Based on the AJCC 7th TNM classification.

Group 1: <65 years, young age; Group 2: 65–74 years, elderly age; Group 3: ≥75 years, very elderly age.

### Postoperative surgical outcomes

Table 2 shows the postoperative surgical outcomes. There were no statistical differences among the three groups except for retrieved lymph node and median follow-up duration (*P* = 0.002, *P* = 0.033), respectively. Further, the median follow-up duration of group 3 was shorter than in the case of the other groups.

**Table 2:** Postoperative outcomes

	**Group 1 (n = 672)**	**Group 2 (n = 285)**	**Group 3 (n = 98)**	***P *value**
Operative time (minutes)*	204.0 ± 55.5	204.8 ± 60.4	193.1 ± 62.1	0.183
Hospital stay (days)*				
with complication	7.9 ± 6.9	7.7 ± 5.4	9.2 ± 9.5	0.133
without complication	7.2 ± 2.1	7.0 ± 1.5	7.2 ± 1.3	0.474
**Type of gastrectomy**				0.693
Total	48	24	6	
Subtotal	624	261	92	
**Reconstruction**				0.458
Billroth-I	369	173	55	
Billroth-II	246	87	36	
Roux-en-Y	57	25	7	
**Lymph node dissection**				0.461
D1+	33	19	4	
D2	639	266	94	
Retrieved lymph node (number)^*^	37.5 ± 13.9	34.7 ± 14.2	33.5 ± 12.3	0.002
First flatus time (days)^*^	2.8 ± 1.1	3.0 ± 1.1	2.9 ± 1.1	0.209
**Post-operative transfusion**	20	8	6	0.231
**Median follow-up duration (months, range)**	52.1 (1.5–136.0)	52.3 (0.7–135.3)	42.7 (4.7–117.4)	0.033

*All values are the means and standard deviations.

Group 1: <65 years, young age; Group 2: 65–74 years, elderly age; Group 3: ≥75 years, very elderly age.

### Postoperative morbidity and mortality

In Table 3, group 1 had 62 complications after surgery (9.2%), group 2 had 26 complications (9.1%), and group 3 had 18 complications (18.4%). In group 1, there were 5 major complications requiring re-operation (1 intra-abdominal abscess, 4 duodenal stump leakages). There were 2 major complications with re-operation (1 duodenal stump leakage, 1 esophagojejunostomy leakage) and 2 mortalities (1 multi-organ failure, 1 duodenal stump leakage) in group 2. In the case of the mortality with multi-organ failure, death occurred because of multi-organ failure after 28 postoperative days. This patient had a very complicated pre-operative history, with underlying pneumonia, atrial fibrillation, right middle cerebral artery infarction, and hypertension. He underwent laparoscopy-assisted distal gastrectomy (Billroth-I). After surgery, his condition necessitated removal to the intensive care unit; however, unfortunately, his cardiac, pulmonary, and renal functions gradually failed.

**Table 3:** Postoperative complications among the three groups

	**Group 1 (n = 672)**	**Group 2 (n = 285)**	**Group 3 (n = 98)**	***P *value**
Complications (%)	62 (9.2)	26 (9.1)	18 (18.4)	0.008
Wound infection	6	5	3	
Intra-abdominal abscess	6 (1*)	1	0	
Intra-abdominal bleeding	6	3	0	
Intra-luminal bleeding	12	3	5	
Postoperative ileus	9	2	2	
Bile leakage	0	1	0	
Duodenal stump leakage	5 (4^*^)	2 (1^*, **^)	1 (1^*^)	
Esophagojejunostomy leakage		1		
Acute pancreatitis	2	0	0	
Pulmonary disease	9	4	1	
Urinary complication	2	1	3	
Hepatic complication	0	1	0	
Cardiac disease	0	0	1	
A-loop syndrome	0	0	1	
Multi-organ failure		1 (1^**^)	0	
Others	5	1	1	

*Reoperation; **Mortality.

Group 1: <65 years, young age; Group 2: 65–74 years, elderly age; Group 3: ≥75 years, very elderly age.

There was a statistical difference among the three groups (*P* = 0.008) in terms of post-operative complications. Group 3 had the highest proportion (18.4%) of postoperative complications of the three groups. In group 3, there was one major complication (duodenal stump leakage) requiring re-operation. However, according to the Clavien-Dindo classification used to assess the severity and incidence of post-operative complications, there was no significant difference in minor and major complications among the three groups (*P* = 0.562) (Table 4).

**Table 4:** Postoperative complications by the Clavien-Dindo classification

	**Group 1 (n = 672)**	**Group 2 (n = 285)**	**Group 3 (n = 98)**	***P *value**
**Minor complications**				0.114
Grade I	31	11	6	
Grade II	18	10	7	
**Major complications**				0.113
Grade III-a	7	1	2	
Grade III-b	5	2	1	
Grade IV-a	1	0	2	
Grade IV-b	0	0	0	
Grade V	0	2	0	

Group 1: <65 years, young age; Group 2: 65–74 years, elderly age; Group 3: ≥75 years, very elderly age.

## Discussion

The average lifespan of Koreans is currently 74.4 years for men and 81.8 years for women. In 2030, the percentage of the population aged 65 and older in Korea is projected to exceed 20% [19]. Gastric cancer is more prevalent in Korea than in western countries. Early gastric cancer is also found more frequently in Korea because of a well-developed mass-screening system [4,5]. In addition, laparoscopic procedures for early gastric cancer have been widely accepted in Korea [5]. The goals of laparoscopic surgery for gastric cancer are to minimize surgical insults and to maximize the patient’s quality of life, while preserving the extent to which the tumor is removed and the appropriate negative margins achieved. Previous reports have demonstrated excellent short-term outcomes including less post-operative pain, improved cosmesis, early recovery, and improved quality of life [6-8].

Elderly patients are more likely to have concurrent medical conditions than are younger patients. Previous studies have shown that, as a result of comorbidity and decreased functional reserves, open gastric surgery in elderly patients is associated with increased morbidity and mortality rates, and a longer hospital stay [20,21]. In a study of laparoscopic cholecystectomy, the post-operative complication and mortality rates were higher in elderly patients than in younger patients, and more comorbidities among elderly patients, especially coronary artery disease, accounted for this adverse outcome [22]. In a multicenter study of colorectal cancer, 85% of the elderly group had at least one cardiovascular risk factor [23]. In our study, we observed similar results with the proportion of comorbidities increasing as the age increased (*P* <0.001).

Life expectancy has been increasing, and recently, numbers of elderly patients with gastric cancer who are considered as candidates for LAG are expanding. However, the safety of LAG in elderly patients has not been proved because of the possible adverse hemodynamic and respiratory effects of the pneumoperitoneum on the limited cardiopulmonary reserve of these patients [24,25]. Pre-operative comorbidities were observed frequently in elderly patients undergoing conventional open gastrectomy [26,27]. The incidence of post-operative complications following open gastrectomy in elderly patients was also reported more frequently than in younger patients [26,27]. However, many reports have concluded that LAG is a feasible and safe procedure in elderly patients if the patients have been selected carefully and the procedure is performed by an experienced laparoscopic surgeon [28-30].

In the present study, although the number of retrieved lymph nodes in group 3 was less than in the other two groups (*P* = 0.002), the extent of lymph node dissection was not of statistical significance among the three groups (*P* = 0.461). Also, the median follow-up period in this group was shorter than in the other two groups (*P* = 0.033). The results reflect the fact that LAG is a more recently performed procedure, and more experience is being gained with laparoscopic surgery in very elderly patients. Nevertheless, despite these findings, the proportion of comorbidities increased as the age increased among the three groups. Further, our results showed that patients in group 3 (≥75 years) frequently had a higher rate of postoperative complications than other groups. However, in order to assess the severity and incidence of postoperative complications among the three groups, we standardized and graded the postoperative complications according to the Clavien-Dindo classification system [17,18]. According to the Clavien-Dindo classification system, there was no statistically significant difference among the three groups. Moreover, when post-operative complications were considered, there was no statistically significant difference in post-operative transfusion rates (*P* = 0.231).

There have been many reports of LAG in elderly patients. However, most reports analyzed the post-operative complications in terms of the rate of complications [10,11,26,29,30]. In the present study, we considered the rate and severity of post-operative complications among the three groups. As a result, our retrospective study demonstrated that the post-operative complications occurred more frequently in very elderly patients undergoing LAG. However, in terms of the severity of postoperative complications for LAG in elderly and very elderly patients, there was no difference compared to other groups. These results showed that most of the postoperative complications in elderly and very elderly patients are minor complications according to the Clavien-Dindo classification system, which may be managed by conservative treatment with their age status taken into account.

Although this study is a retrospective study, with data prospectively collected and reviewed from the gastric cancer database of our institute, we propose that, in respect of the severity of post-operative complications, the post-operative complications of elderly and very elderly patients with gastric cancer who undergo LAG are no different than those in younger age groups.

## Conclusions

Elderly patients undergoing gastric cancer operations are exposed to potential operative risks and may experience post-operative morbidity and mortality. Although the rate of post-operative complications in very elderly patients who undergo LAG may be relatively high, the severity of postoperative complications is of a similar degree to that of other age groups. In analyzing any study of postoperative complications in elderly patients who have undergone any operations, not only the rate of postoperative complications, but also the severity of postoperative complications, must be considered.

## Abbreviations

LAG: Laparoscopy-assisted gastrectomy; BMI: Body mass index.

## Competing interests

The authors declare that they have no competing interest.

## Authors’ contributions

KH Kim carried out data collection. MC Kim and GJ Jung helped draft the manuscript. All authors read and approved the final manuscript.
